# Selective Enrichment Media Bias the Types of *Salmonella enterica* Strains Isolated from Mixed Strain Cultures and Complex Enrichment Broths

**DOI:** 10.1371/journal.pone.0034722

**Published:** 2012-04-04

**Authors:** Lisa Gorski

**Affiliations:** Produce Safety and Microbiology Research Unit, United States Department of Agriculture, Agricultural Research Service, Albany, California, United States of America; Charité-University Medicine Berlin, Germany

## Abstract

For foodborne outbreak investigations it can be difficult to isolate the relevant strain from food and/or environmental sources. If the sample is contaminated by more than one strain of the pathogen the relevant strain might be missed. In this study mixed cultures of *Salmonella enterica* were grown in one set of standard enrichment media to see if culture bias patterns emerged. Nineteen strains representing four serogroups and ten serotypes were compared in four-strain mixtures in *Salmonella*-only and in cattle fecal culture enrichment backgrounds using *Salmonella* enrichment media. One or more strain(s) emerged as dominant in each mixture. No serotype was most fit, but strains of serogroups C2 and E were more likely to dominate enrichment culture mixtures than strains of serogroups B or C1. Different versions of Rappaport-Vassiliadis (RV) medium gave different patterns of strain dominance in both *Salmonella*-only and fecal enrichment culture backgrounds. The fittest strains belonged to serogroups C1, C2, and E, and included strains of *S.* Infantis, *S.* Thompson *S.* Newport, *S.* 6,8:d:-, and *S.* Give. Strains of serogroup B, which included serotypes often seen in outbreaks such as *S.* Typhimurium, *S.* Saintpaul, and *S.* Schwarzengrund were less likely to emerge as dominant strains in the mixtures when using standard RV as part of the enrichment. Using a more nutrient-rich version of RV as part of the protocol led to a different pattern of strains emerging, however some were still present in very low numbers in the resulting population. These results indicate that outbreak investigations of food and/or other environmental samples should include multiple enrichment protocols to ensure isolation of target strains of *Salmonella*.

## Introduction

Enrichment culture is a competition among microbiota for available nutrients and against growth inhibitors. While enrichment media are designed to favor a target organism the conditions may not favor equally every strain or sub-group (e.g. serotype, serogroup) of that species. This is of particular concern when the organism being sought is a pathogen from a complex matrix, such as a foodborne pathogen. The issue of culture bias or culture fitness between strains of the same species or subgroup has been described for *Listeria monocytogenes* [1], [2] and *Salmonella* [3], [4]. Natural variants present in *Escherichia coli* O157:H7 populations show major differences in stress resistance that affect the phenotypes isolated [5].

Ongoing projects in the lab include surveys for the prevalence and diversity of *Salmonella* in native and agricultural environments of California [6]. During sample processing and enrichment often only one strain of *Salmonella* is isolated per sample; however, on occasion more than one strain (as determined by pulsed field gel electrophoresis) was isolated from the same sample (L. Gorski, unpublished data). These results have stimulated questions about the efficiency of recovery of *Salmonella* from enrichment media containing more than one strain of *Salmonella* and whether a particular enrichment protocol might bias the types of strains isolated (serotype, serogroup, genotype, etc). Enrichment bias based on serotype has been reported with some protocols for *L. monocytogenes* [1], [2], so it was natural to ask if serotype or serogroup influenced potential enrichment fitness or bias in *Salmonella*.

Many enrichment protocols and media have been described for the isolation of *Salmonella*, and often include variations on Rappaport-Vassiliadis Medium (RV) [7], which is recommended in the Food and Drug Administration Bacterial Analytical Manual enrichment protocol for *Salmonella* [8]. In past and ongoing survey projects RV, Rappaport-Vassiliadis Soya Peptone Broth (RVS), and Modified Semi-Solid Rappaport-Vassiliadis Medium have been or are used in conjunction with Xylose Lysine Desoxycholate Agar [6]. Previous studies reported that different *Salmonella* strains have different recovery characteristics in enrichment media leading to differential recovery of one strain over another [3], [4], but no one has asked if these differences were related to serotype or serogroup. This information is vital for accurate surveillance and outbreak investigations for traceback studies to identify pathogen reservoirs and point sources of contamination of food or water. In the present study the competition between multiple *Salmonella* strains present in the same mixture was assessed. Nineteen different *Salmonella* strains representing four different serogroups and ten different serotypes were tested in different *Salmonella* enrichment protocols either with *Salmonella*-only cultures or mixtures of *Salmonella* in a fecal enrichment culture background.

## Materials and Methods

### Bacterial Cultures, Media, and Culture Conditions

Strains of *S. enterica* used in this study are in Table 1. For routine use cultures were grown in Trypticase Soy Broth (TSB, Millennium Laboratories, Anaheim, CA) or on TSA plates (TSB solidified with 1.5% agar) at 37°C. RV Medium was prepared according to the FDA Bacterial Analytical Methods manual [8]. RVS Broth was from Oxoid (Basingstoke, Hampshire, England). Xylose Lysine Desoxycholate Agar (XLD) was from Difco (Becton Dickinson, Franklin Lakes, NJ). Serial dilutions were made in Phosphate Buffered Saline (PBS, 10 mM sodium phosphate pH 7.2, 150 mM NaCl).

**Table 1:** Strains used in this study.

Strain	Serogroup	Serotype	Source
RM2519	B	Typhimurium	Human
RM7910	B	Saintpaul	Human (jalapeno pepper outbreak)
RM10602	B	Typhimurium	Water
RM10608	B	Typhimurium	Bird
RM14122	B	Schwarzengrund	Bird
RM14130	B	Saintpaul	Water
RM1987	C1	Thompson	human (cilantro outbreak)
RM11056	C1	Infantis	Skunk
RM11481	C1	Infantis	Hog
RM14128	C1	Thompson	Water
RM14398	C1	Thompson	Chard
RM1655	C2	Newport	Alfalfa (outbreak)
RM10604	C2	6,8:d:-	Water
RM11772	C2	Kentucky	Pig
RM14111	C2	Newport	squirrel
RM10601	E	Give	Water
RM10965	E	Give	Soil
RM14106	E	Uganda	squirrel
RM14126	E	Uganda	Water

### Strain Selection and Preparation of Enrichment Cultures

Mixtures of *Salmonella* were designed so they contained four strains representing serogroups B, C1, C2, and E. The make-up of these mixtures is shown in Table 2. Growth rates of the strains in TSB at 37°C were measured in a Bioscreen C (Growth Curves, USA, Piscataway, NJ) with readings every 15 minutes over a 24 h period. Strains of similar growth rates were selected based on serogroup and serotype to represent human clinical and environmental isolates of serotypes similar to those identified in a 2008–2009 survey of the central California leafy greens production region in for the incidence of *Salmonella* [6], as well as more recent isolates acquired from California wildlife and water samples collected in 2010 and 2011 (L. Gorski, unpublished data).

**Table 2:** Dominant strains from RV and RVS cultures after enrichment process.

Mixture	Strain	Serotype	Final cell density from RV^a^	Dominant strain(s) and serogroups in mixture from RV^b^	Dominant strain (s) and serogroup(s) in mixture from RVS^b^
1	RM2519	Typhimurium	6.92±0.11	RM11056, C1	RM2519, B
	RM11056	Infantis	6.91±0.16		
	RM1655	Newport	6.66±0.46		
	RM14106	Uganda	6.37±0.17		
2	RM7910	Saintpaul	6.06±0.02	RM10601, E	RM11772, C2RM10601, E
	RM11481	Infantis	7.06±0.10		
	RM11772	Kentucky	6.67±0.06		
	RM10601	Give	7.60±0.41		
3	RM10602	Typhimurium	6.07±0.16	RM14128, C1RM10601, E	RM14128, C1RM1655, C2RM10601, E
	RM14128	Thompson	7.21±0.47		
	RM1655	Newport	6.66±0.46		
	RM10601	Give	7.60±0.41		
4	RM14122	Schwarzengrund	6.69±0.01	RM10604, C2	RM10604, C2RM14126, E
	RM14398	Thompson	6.82±0.06		
	RM10604	6,8:d:-	8.22±0.05		
	RM14126	Uganda	6.14±0.47		
5	RM14130	Saintpaul	7.43±0.13	RM14130, BRM10965, E	RM14111, C2RM10965, E
	RM1987	Thompson	6.64±0.02		
	RM14111	Newport	7.36±0.04		
	RM10965	Give	6.92±0.01		
6	RM10608	Typhimurium	7.93±0.05	RM10608, BRM14111, C2	RM14126, E
	RM14398	Thompson	6.82±0.06		
	RM14111	Newport	7.36±0.04		
	RM14126	Uganda	6.14±0.47		
7	RM10608	Typhimurium	7.93±0.05	RM10604, C2	RM10604, C2
	RM14128	Thompson	7.21±0.47		
	RM10604	6,8:d:-	8.22±0.05		
	RM10601	Give	7.60±0.41		
8	RM10608	Typhimurium	7.93±0.05	RM10601, E	RM1655, C2RM10601, E
	RM1987	Thompson	6.64±0.02		
	RM1655	Newport	6.66±0.46		
	RM10601	Give	7.60±0.41		

a log CFU/ml of individual strain grown in RV at 42°C.

b Dominant strain(s) determined by highest cell density and ratio of strain in multiple experiments.

Cultures were grown overnight at 37°C in TSB and diluted 1∶100 the following morning and grown to early to mid-log phase. These cultures were diluted in PBS to an A_600_ of 0.2 (∼1.5×10^8^ CFU/ml), and the suspensions were dilution plated onto TSA to calculate cell concentrations. One hundred microliters of 10^−4^ dilutions of the cell suspensions in PBS were mixed together in a tube, and 100 µl of these 1∶1∶1∶1 cell suspensions were inoculated into duplicate flasks of 25 ml TSB for a starting combined concentration of ∼24 CFU/ml. The enrichment protocol from the FDA Bacteriological Analytical Manual [8] was followed. Briefly the TSB cultures were incubated at 35°C in a rotating shaker at 150 rpm for approximately 24 h. One hundred microliters of the resulting mixed TSB culture was inoculated into 10 ml of RV and/or RVS broth in 16×150 mm test tubes that were inserted into slanted racks and incubated at 42°C in a shaking incubator for 24 h. The resulting cultures were dilution plated onto XLD agar, and incubated at 37°C overnight. XLD plates with 30–500 colonies from each RV or RVS culture were used for immunoblotting (see below). For experiments measuring strain distribution in the TSB mixed cultures, dilutions were plated onto TSA plates and incubated at 37°C for 18 h before immunoblotting (see below). All experiments measuring the different conditions were performed at least twice.

A cattle fecal enrichment culture was used as background microbiota in some experiments. This culture was made from 10 g of cattle feces collected from the ground at a ranch in Monterey County, CA and suspended in 90 ml of TSB. This culture was incubated at 25°C for 2 h with shaking at 200 rpm followed by 8 h at 42°C at 200 rpm, and holding at 4°C until the following morning [6], [9]. Sterile glycerol was added to a final concentration of 1 M and aliquots were frozen at −80°C. For experiments evaluating *Salmonella* in background cattle fecal microbiota, approximately 100 µl of this frozen suspension was inoculated into 10 ml of TSB and grown overnight at 35°C. This culture was enumerated by dilution plating onto TSA, which was incubated at 37°C. A 10^−4^ dilution of the fecal culture was made and 100 µl of this dilution was added to the 25 ml TSB cultures inoculated with the *Salmonella* mixtures described above. This resulted in a concentration of ∼2800 CFU/ml of cattle fecal bacteria in the enrichment cultures. The fecal culture was inoculated at levels 100 times higher than the *Salmonella* in these experiments to better reflect a natural contamination of a complex sample. The resulting TSB cultures, designated F-TSB, were subcultured into both RV and RVS, and are designated F-RV and F-RVS. The F-RV and F-RVS cultures were dilution plated onto XLD for immunoblotting and to determine *Salmonella* CFU/ml, and onto TSA to determine total aerobic, mesophilic CFU/ml.

### Differentiation of serogroups by immunoblotting

TSA or XLD plates were numbered 1–12, the colonies counted, and transferred by colony lift for 10 min onto 0.45 µm pore size nitrocellulose Protran BA85 membrane 82 mm circles (Whatman, Piscataway, NJ). Each of the membranes was pre-labeled with the plate number to keep track of the number of colonies blotted. All membrane incubations were done at room temperature on a bench top shaker. Membranes were washed twice for 10 min each in Wash Buffer (0.1% Tween 20, 150 mM NaCl, 10 mM Tris-HCl, pH 7.5), followed by a 20 min incubation in Wash Buffer at 80°C to kill pathogens. Membranes then were incubated in Blocking Solution (0.5% Casein, 30 mM NaN_3_, 150 mM NaCl, 10 mM Tris-HCl, pH 7.5) for at least 1 h, followed by incubation with primary antiserum (see below) in Dilution Buffer (1% Bovine Serum Albumin, 2.7 mM KCl, 15 mM NaN_3_, 0.1% Tween 20, 150 mM NaCl, 10 mM Tris-HCl, pH 7.5) for 30–40 min. Membranes were washed twice in Wash Buffer for 10 min each, incubated with a 1∶30,000 dilution of secondary antibody (Alkaline phosphatase-conjugated goat anti-rabbit IgG, Sigma-Aldrich, St. Louis, MO) in Dilution Buffer for 45–90 min, and washed again with two 10 min incubations in Wash Buffer. Colonies were visualized with either SIGMA*FAST* BCIP(R)/NBT (Sigma-Aldrich) or BCIP/NBT Color Development Substrate (Promega, Madison, WI) according to manufacturer's instructions.

Anti-*Salmonella* O Antiserum for Groups B, C1, C2, and E (Difco), *Salmonella* O Antiserum Factor 7 (for Group C1, Denka Seiken, Tokyo, Japan) and Factor 8 (for Group C2, Difco) were used to identify colonies of these serogroups. All of the *Salmonella* strains were screened with all of the antisera initially by ELISA as described [10] to determine cross-reactivity of the antisera and the appropriate dilutions for specific identification of serogroups. Only one *Salmonella* antisera reacted across multiple serogroups in immunoblots, but the antiserum bound higher to the target serogroup than background binding to other serogroups, so it was useable in immunoblots. *Salmonella* O antisera were used as primary antisera in immunoblots at dilutions ranging from 1∶500 to 1∶2000.

Each membrane was pre-labeled with the primary antiserum used. After color development blue colonies on each of the blots were counted and compared to the total number of colonies on the original plate to determine a ratio of the population corresponding to a given serogroup.

### Statistics

Each enrichment was cultured in duplicate and plated onto 12 plates resulting in 24 plates per mixture per experiment. Each set of 12 blots was divided into 4 sets of triplicates for incubation with each of the 4 primary *Salmonella* antisera. All experiments were performed at least twice. Statistics were done in Microsoft Excel 2007.

## Results

### Culture bias in RV enrichments

**Figure 1:**
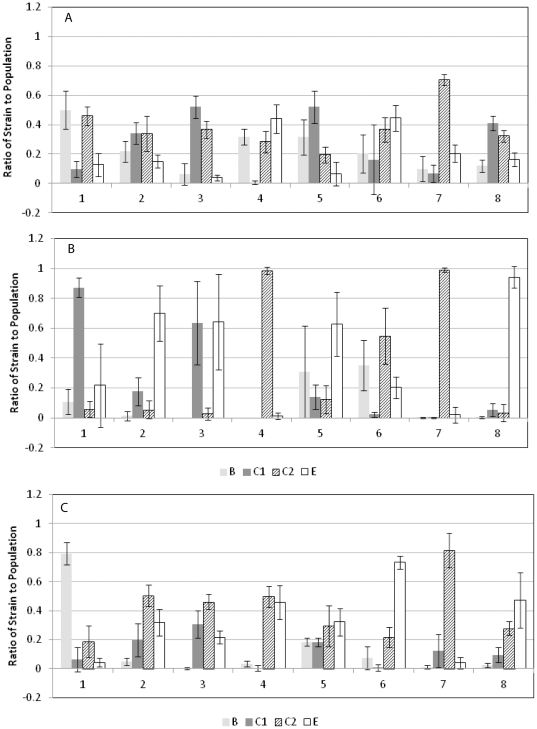
Ratio of each strain in the *Salmonella* enrichment to the whole population. (A) Ratios from the TSB mixtures plated onto TSA agar; (B) Ratios from enrichments using RV and plated onto XLD; (C) Ratios from enrichments using RVS and plated onto XLD. Values are the averages of all replicates from multiple experiments, and the error bars represent standard deviation.

Strains were sorted into eight groups of four based on similar growth rates in TSB, final cell density in enrichments using RV medium, and those that grew best in RV individually. Each strain had equivalent growth kinetics in TSB mono-culture at 37°C (data not shown); however bias was already noticeable after the first step in the enrichment process, non-selective growth in TSB (Figure 1A). While these TSB enrichment cultures were inoculated with equivalent numbers of each strain the ratios were significantly changed in the resulting cultures. The ratios of each strain in each mixture after growth in TSB, subculturing in RV, and plating onto XLD are shown in Figure 1B and the results are summarized in Table 2. The strains that dominated the TSB cultures did not necessarily reflect those that dominated the RV cultures inoculated from them (Figures 1A and 1B). For example the group C1 strain in Mixture 1 was approximately 10% of the TSB culture but then comprised 90% of the RV culture resulting from it (Figures 1A and 1B). Also the group E strains that dominated the RV cultures in Mixtures 2, 3, 5, and 8 comprised less than 20% of the inocula from TSB. Strains from serogroups C1, C2, and E dominated all the enrichments from RV with serogroup C1 strains dominating in two of the mixtures, serogroup C2 strains dominating in three of the mixtures, and serogroup E strains dominating in four of the strain mixtures. Strains from serogroup B never dominated an RV culture by themselves, but co-dominated in Mixtures 5 and 6.

Table 3 shows the final *Salmonella* cell densities for the enrichment mixtures in the succession of enrichment media (TSB, and TSB followed by RV or RVS). Cell densities from RV after growth in ranged from 6.56 log CFU/ml to 8.16 log CFU/ml. Because of this 2-log variability the final cell density of each individual strain in RV mono-culture was measured by dilution plating onto XLD (Table 2). The range of final cell densities from these individual RV cultures also had a 2-log range (6.06–8.22 log CFU/ml), and was not significantly different among the serogroups (*P*>0.05). The strains with the highest cell densities in RV in mono-culture dominated seven of the eight *Salmonella* multi-cultures after growth in the succession of TSB, RV, and XLD. The exception was Mixture 8 where the group B strain RM10608 had the best individual growth in RV, but the group E strain RM10601 dominated. In the three mixtures that had two dominant strains (Mixtures 3, 5, and 6) the two strains that grew best in RV mono-culture were dominant in Mixtures 3 and 6. The second best individual RV grower in Mixture 5 was the group C2 strain RM14111, but it was outcompeted in the enrichment mixture by the group E strain RM10965, the third best grower in RV mono-culture.

**Table 3:** Final cell densities (log CFU/ml) of the mixtures in TSB, RV, and RVS medium.

Culture Media Succession	Plating medium	Mixture							
		1	2	3	4	5	6	7	8
TSB^a^	TSA	9.93±0.04	9.95±0.06	10.00±0.05	9.84±0.06	9.98±0.6	9.83±0.04	9.93±0.08	10.20±0.04
TSB→RV^a^	XLD	7.46±0.06	7.84±0.13	7.23±0.20	8.16±0.05	6.56±0.57	7.27±0.23	8.01±0.20	7.56±0.13
TSB→RVS^a^	XLD	8.92±0.09	8.84±0.08	8.80±0.07	8.91±0.05	8.92±0.02	8.88±0.05	8.86±0.10	9.01±0.18
F-TSB→F-RV^b^	XLD	7.30±1.07	7.32±0.97	7.26±0.86	8.43±0.03	6.74±1.05	7.33±1.24	8.32±0.12	8.13±0.04
F-TSB→F-RVS^b^	XLD	8.94±0.13	8.99±0.06	8.76±0.07	8.93±0.07	8.92±0.10	8.81±0.15	8.73±0.12	8.89±0.09
F-TSB→F-RV^b^	TSA	8.14±0.09	8.09±0.03	8.44±0.11	8.59±0.03	8.23±0.10	8.32±0.01	8.45±0.09	8.19±0.01
F-TSB→F-RVS^b^	TSA	9.04±0.06	9.04±0.12	8.89±0.17	8.93±0.23	8.96±0.01	8.94±0.03	8.90±0.04	9.06±0.10

a *Salmonella*-only cultures.

b These cultures contained cattle fecal bacteria with added *Salmonella*.

### Culture bias in enrichments using RVS

These results stimulated additional experiments with another commonly used enrichment medium. RVS has more nutrients than RV resulting in better growth of individual *Salmonella* strains. The final cell densities of mono-cultures of the strains were higher in RVS and more uniform than RV, ranging from 8.6 log CFU/ml to 9 CFU/ml. Similarly, as shown in Table 3, the final cell densities of the strain mixtures after growth in TSB and subculturing in RVS were higher and more equivalent than those from RV (8.8 log CFU/ml to 9.01 log CFU/ml). Inoculation of the enriched TSB samples into RVS and plating on XLD resulted in different strain distributions compared to RV (Figure 1C, Table 2). The strain distributions in each mixture were more equivalent from the RVS cultures and some strains that were barely detectable in some of the mixtures from RV became dominant or co-dominant in RVS. For example, the group B strain RM2519 in Mixture 1 made up 10% of the enrichment using RV, but it comprised 80% of the population when the enrichment used RVS. Similarly RM14126, the group E strain in Mixture 4 was barely detectable in the RV enrichments, but was co-dominant in RVS. The serogroup distributions differed from those from RV, but serogroups C2 and E were dominant in most of the mixtures with groups C2 and E strains each dominating in 6 of the mixtures (Table 2). The strain distributions from RVS were similar to the distributions after growth of the mixtures in TSB (Figure 1A), but there were subtle differences between the two conditions.

### Effect of background microbiota on *Salmonella* strain distribution in enrichments

A fecal enrichment culture from cattle was used to provide a population of background microbiota to see if the biases seen with *Salmonella*-only enrichments were affected by a complex mixture of fecal microflora. The fecal enrichment culture was negative for *Salmonella* by the enrichment protocols used in this study. The levels of total aerobic, mesophilic growth were less from F-RV (8.09–8.59 log CFU/ml) than from F-RVS (8.89–9.06 log CFU/ml), consistent with the better growth of *Salmonella* strains in RVS compared with RV. Similarly levels of *Salmonella*, as measured by black colonies on XLD, were lower from F-RV cultures (6.74–8.32 log CFU/ml) than from F-RVS cultures (8.73–8.99 log CFU/ml). The F-RV cultures showed a nearly 2-log range in final cell density as measured on XLD plates, similar to the RV mixtures that contained only *Salmonella*. The RV and F-RV cultures were roughly equivalent in their CFU/ml as measured on XLD, as were the RVS and F-RVS cultures, indicating that the background microbiota did not affect the growth of total *Salmonella* in the mixtures.

**Figure 2:**
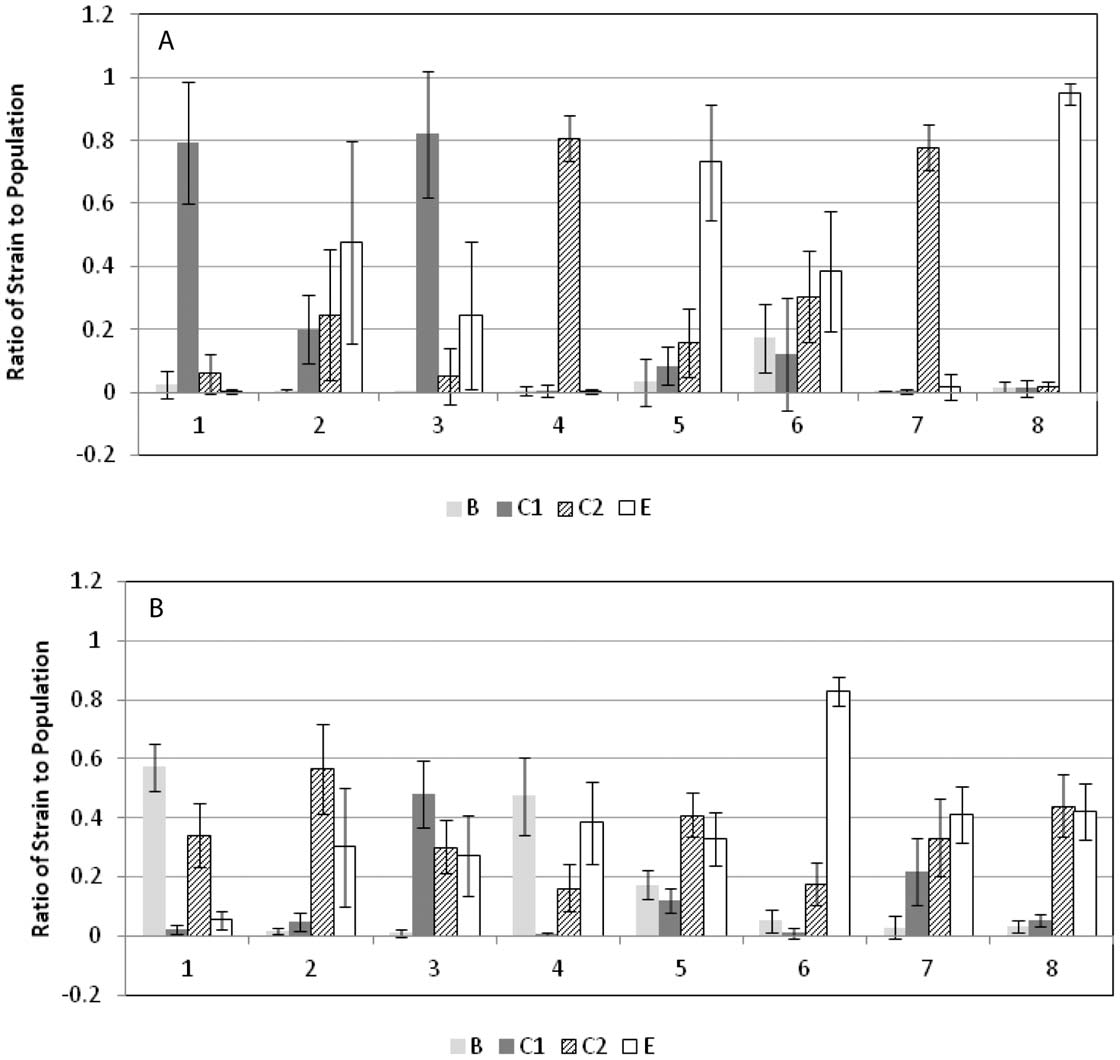
Ratio of each strain of *Salmonella* in the *Salmonella*+fecal bacteria enrichments to the whole *Salmonella* population. (A) Ratios from enrichments using RV and plated onto XLD; (B) Ratios from enrichments using RVS and plated onto XLD. Values are the averages of all replicates from multiple experiments, and the error bars represent standard deviation.

The distribution of the *Salmonella* strains after enrichment in TSB and RV or RVS in the presence of the fecal culture is shown in Figure 2. Because of the high levels of background bacteria immunoblots from TSA plates of F-TSB cultures could not be done. Similar to the experiments with the *Salmonella*-only enrichments the distribution differed between F-RV and F-RVS, with F-RV displaying greater differences between the strains in each mixture. In comparing RV (Figure 1B) with F-RV (Figure 2A) there were few differences in the dominant strains in each mixture. The group C1 strain, RM14128, was present in a higher ratio in the F-RV than the RV enrichments in Mixture 3, and the distribution in Mixture 6 was more equivalent from F-RV than the RV enrichments. Strains from serogroups C1, C2 and E each dominated in four of the F-RV enrichment mixtures. For both the RV and F-RV enrichments the serogroup B strain was consistently detectable only in Mixture 5, which had the lowest total *Salmonella* growth as measured by plating on XLD (Table 3). Comparing the F-RVS and RVS conditions there was less variability among the distribution of the *Salmonella* strains resulting from F-RVS. The group E strain RM14126 clearly dominated Mixture 6 from RVS and F-RVS, but the rest of the mixtures showed a more equivalent distribution among the co-dominant strains in the F-RVS enrichments. Two strains that showed less dominance in F-RVS vs RVS were the group B strain RM2519 and the group C2 strain RM10604 in Mixtures 1 and 7, respectively. Some strains were present in very low ratios from F-RVS including many of the serogroup B and C1 strains. Exceptions were the group B strains RM2519 and RM14122 in Mixtures 1 and 4, respectively, and the group C1 strain RM14128 in Mixture 3, all of which were among the dominant strains in F-RVS. Group B strain RM14122 (Mixture 4) performed better in the presence of the fecal microbiota in F-RVS than it did in the presence of only other *Salmonella* strains in RVS, suggesting the fecal microbiota may contribute factors that are advantageous to the growth of some strains of *Salmonella* (Figures 1C and 2B). Strains from serogroup C2 dominated in five of the F-RVS mixtures, and group E strains dominated in six of the mixtures.

## Discussion

More than 2500 serotypes of *Salmonella* have been identified, yet most human infections are traced to only a small fraction of that number [11]–[13]. The most common serotypes in human illness in the United States in 2009 included the serotypes (with serogroup in parentheses) Enteritidis (D1), Typhimurium (B), Newport (C2), Javiana (D1), Saintpaul (B), Infantis (C1), Thompson (C1), and Schwarzengrund (B). The list of serotypes isolated most often from non-clinical, non-human sources differs from those implicated in illness. Non-human, non-clinical serotypes (and serogroups) isolated most frequently in 2009 included Kentucky (C2), Enteritidis (D1), Heidelberg (B), Typhimurium (B), Senftenberg (E4), and Hadar (C2) [13]. Also the serotype distribution in disease and environmental cases differs based on geography [6], [12]–[15]. In a recent survey of a California agricultural environment, which included sampling of livestock, water, soil, and wildlife, the most commonly isolated serotypes (and serogroups) were Give (E1), Typhimurium (B), and the monophasic types 6,8:d:- (C2), and 6,8:-:e,n,z_15_ (C2) [6]. Ongoing surveys of additional samples from California have resulted in the isolation of more of the above plus additional serotypes (L. Gorski, unpublished data). These differences in serotypes and serogroups isolated from different sources may reflect the actual ecology of the environments; however, it may also reflect culture biases in the enrichment protocols used to isolate them.

Many enrichment protocols with different media and temperatures are used for the enrichment of *Salmonella* from various sources. In this study the focus was on the enrichment protocol using variations of RV medium from TSB non-selective primary enrichments. This is a common protocol used in the lab for surveys from produce, soil, water, wildlife, and cattle, and variations of it are given in the FDA-BAM Manual [8] and the Environmental Protection Agency method for monitoring of *Salmonella* in water and biosolids [16]. XLD was used as a detection medium for *Salmonella* because it is more likely to yield black colonies from a wide variety of *Salmonella* isolates [6]. Different protocols and media may give different strain fitness profiles. Also in this artificial enrichment environment the *Salmonella* strains were inoculated in equal proportions, which is unlikely in a multiple strain contaminated sample. Other fitness studies have shown that dominant strains tend to continue to dominate mixtures even when inoculation ratios were varied [3].

The comparison of *Salmonella* strains based on serogroup was addressed for two reasons. First, O group-specific antiserum to differentiate strains was available. Second, strains can be grouped genetically based on differences in the LPS biosynthetic genes which encode the O-group antigens in most cases encoded by the *rfb* genes on the genome [17], [18]. *S.* Kentucky is the most commonly isolated serotype from non-human, non-clinical sources [13], and the question has been raised whether *S.* Kentucky or other strains in the C2 serogroup are more prevalent in the environment or simply more fit under enrichment culture conditions used in surveys. Studies showed that *L. monocytogenes* strains of serotype 1/2a were fitter than serotype 4b strains in one standard enrichment protocol [2], while another study found no evidence of culture bias associated with serotype using a second standard enrichment method [1]. It is likely that a *Salmonella* enrichment protocol different from the one used in the present study would give different fitness results. Statistical analysis of a *S.* Enteritidis strain concluded that the performance of the strain in enrichment culture was dependent on the enrichment protocol used [3].

The overall difference in strain distributions resulting from the RV and RVS enrichments were not surprising. Others have reported strain-specific differences in enrichment cultures and the use of more than one selective medium to ensure isolation of different types of strains from complex samples [19]–[21]. The best individual performers in RV mono-culture tended to dominate enrichment mixtures that included RV. None of the four serogroups or any serotype always was dominant in all the mixtures and media, but some trends were evident. Strains of serogroups C2 and E tended to be among the dominant strains in the enrichments. This result is in line with the frequent isolations of serotypes Newport and Kentucky reported by the CDC [13], and the frequent isolations in California of serotypes Give and 6,8:d:- [6]. Singer *et al.* [3] found also that an *S.* Newport strain was the fittest of four *Salmonella* strains tested in enrichment protocols. In the present study strains of serogroup B generally were the least fit among the four serogroups. From RV and F-RV enrichments the serogroup B strains were only detectable when the total *Salmonella* count was less than 7 log CFU/ml suggesting that the group B strains could not compete well when the total levels were high. Since serogroup B strains are often involved in illness, this result may have implications in surveys and traceback investigations.

Many factors contribute to fitness in mixed cultures including nutrient composition, culture conditions, and the nature of the competing microbiota. It has been recognized for a long time that many strains of various serogroups of *Salmonella* produce colicin-like bacteriocins that can inhibit bacteria of similar or even the same species [22]–[24]. In mixed cultures *Salmonella* could produce and/or be susceptible to compounds produced by other cells in the culture. Different strains of *Salmonella* might have different reactions to inhibitory compounds, which would result in variable fitness.

Culture bias in the present study was evident during the entire enrichment protocol starting with the TSB cultures with the *Salmonella* strain mixtures. That several strains did not dominate the mixed TSB cultures and went on to dominate the RV and F-RV cultures suggest that different factors affect competition of *Salmonella* strains in TSB and RV. These results are in agreement with Singer *et al* [3] who found a serogroup C2 *S.* Newport strain to be most dominant in several enrichment protocols in a study with a mixture of four strains of *Salmonella* with and without added bovine feces, even when the starting concentration of the *S.* Newport strain comprised 10% of the four-strain mixture.

The process of enrichment culture must provide a balance between recovery of the desired organism while avoiding the overgrowth of competing organisms. One reason RV is favored in enrichment protocols is that the combination of inhibitors and the lower nutrient concentration reduces the numbers of competing bacteria such as *Proteus* that might be present in some samples [7]. The addition of soya peptone to RV was shown to increase the growth of *Salmonella* and give slightly better recovery of *Salmonella* in general [25]. In the present study RVS provided a more equivalent distribution of all of the strains than did RV for the mixtures in both the *Salmonella*-only and the added fecal bacteria enrichments.

The results with the fecal enrichment cultures were similar to their corresponding cultures without the added microbiota. This condition represents only one example of the types of competition *Salmonella* would encounter in enrichments, and the distributions of *Salmonella* strains could change depending on the nature of the microbiota present in any sample being tested, in addition to potential chemical or biochemical factors in food and soil matrices routinely in enrichment cultures [26]–[28].

These results emphasize the importance of using multiple enrichment media and methods to increase the probability of isolating *Salmonella* strains that might have different culture-fitness characteristics in the samples being tested. This would be especially critical in outbreak/traceback investigations and environmental surveys that can involve diverse sample types (e.g. water, feces, soil, plants) with diverse native microbiota that could bias the fitness of the target organism. This concern is supported by the results with the serogroup B strains that were detected at the lowest levels of the four groups in this study. The results suggest that a specific target strain should be tested with current methods to determine the best enrichment media and protocol for efficient detection.
